# Attitudes, beliefs and social norms regarding infant and young child feeding among Nigerian mothers, fathers and grandmothers across time

**DOI:** 10.1111/mcn.13524

**Published:** 2023-05-12

**Authors:** Courtney H. Schnefke, Valerie L. Flax, Fred Ubanmhen, Silvia Alayon, Sujata Bose, Obinna Daniel, Kathryn E. L. Grimes, Diana Allotey, Emily R. Seiger, Olujide Arije

**Affiliations:** ^1^ Public Health Research Division RTI International Research Triangle Park North Carolina USA; ^2^ Kantar London UK; ^3^ Department of Global Health Save the Children Washington District of Columbia USA; ^4^ FHI Solutions, Monitoring Learning and Evaluation Durham North Carolina USA; ^5^ RTI International Global Public Health Impact Center; ^6^ Department of Nutrition University of North Carolina‐Chapel Hill Chapel Hill North Carolina USA; ^7^ Institute of Public Health Obafemi Awolowo University Ile‐Ife Osun State Nigeria

**Keywords:** beliefs, breastfeeding, care‐giving, child feeding, complementary feeding, qualitative methods

## Abstract

Infant and young child feeding (IYCF) interventions in low‐resource countries mainly target pregnant women and mothers of young children; however, fathers and grandmothers also influence IYCF practices. We conducted focus group discussions with mothers, fathers and grandmothers of young children across three time points in areas where an IYCF social and behaviour change intervention was implemented in Nigeria to explore differences by participant type and shifts over time in attitudes, beliefs and social norms related to breastfeeding and dietary diversity (DD). Overall, across time points, we found more discrepancies in attitudes, beliefs and social norms for early initiation of breastfeeding (EIBF) and exclusive breastfeeding (EBF) among different participant types than for DD. Although most participants agreed EIBF and EBF are good practices, mothers believed this more strongly than fathers and grandmothers; however, at endline, a shift towards acceptance of EIBF and EBF appeared among fathers and grandmothers. Across time points, all participant types acknowledged the nutritional and health benefits of green leafy vegetables and animal‐source foods but described various barriers to feeding them to children. Across time points, all participant types also highlighted the importance of health workers and antenatal visits as important sources of IYCF knowledge and facilitators to following recommended practices. Insights from this study highlight the importance of including key influencers of IYCF practices in qualitative research.

## Key messages

- Overall, we found more discrepancies in attitudes, beliefs, and social norms for early initiation of and exclusive breastfeeding across participant types than for dietary diversity.
- All types of participants across time points acknowledged the nutritional and health benefits of green leafy vegetables and animal source foods but described various barriers to feeding them to children.
- Across time points, all participant types highlighted the importance of health workers and antenatal visits as important sources of IYCF knowledge and facilitators to following recommended practices.
- This study highlights the importance of expanding the inclusion of key influencers of IYCF practices in future research.

## Graphical abstract

DD.We conducted focus group discussions with mothers, fathers and grandmothers of young children across three time points in areas where an IYCF social and behaviour change intervention was implemented in Nigeria to explore differences by participant type and shifts over time in attitudes, beliefs and social norms related to breastfeeding and dietary diversity (DD). Overall, across time points, we found more discrepancies in attitudes, beliefs and social norms for early initiation of breastfeeding and exclusive breastfeeding among different participant types than for

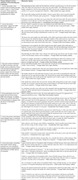

## 1 INTRODUCTION

Optimal infant and young child feeding (IYCF) practices contribute to child survival, health, growth and development (Black et al., 2013; Engle et al., 2007). However, in many low‐ and middle‐income countries (LMICs), the prevalence of optimal breastfeeding and complementary feeding practices remains low (Arabi et al., 2012). Interventions to address this gap usually target pregnant women and mothers of young children, but mothers' child‐feeding decisions and practices do not happen in isolation. Interpersonal relationships and different forms of social support (i.e., emotional, informational and instrumental) can play an important role in influencing health behaviours, including IYCF practices (Standsfeld et al., 2005). For this reason, global recommendations advise including fathers, grandparents and other key nutrition influencers in child nutrition programmes (World Health Organization, et al., 2018).

Evidence on the roles of fathers and grandmothers as key influencers of child‐feeding practices is growing. A recent systematic review found improvements in breastfeeding‐related outcomes, family members' support and family relationships when fathers and grandmothers were included in IYCF interventions, though challenges related to overcoming social norms were also reported (Martin et al., 2020). Research from Senegal (Aubel et al., 2004; Aubel, 2012) and Malawi (Bezner Kerr et al., 2008) describes the role that grandmothers can play in maternal nutrition and child feeding, such as through serving as advisers and caretakers to women and children and emphasises the need to include these ‘culturally designated advisors and caregivers’ in nutrition interventions and programmes. In contrast, some research studies have shown that fathers and grandmothers can have a negative influence (e.g., from inadequate IYCF knowledge, cultural beliefs or pressure to follow or not follow certain practices) or the potential for a negative influence on breastfeeding (Agunbiade & Ogunleye, 2012; Bazzano et al., 2017; Fjeld et al., 2008; Kessler et al., 1995; Rempel & Rempel, 2004; Rempel et al., 2017; Rosane Odeh Susin & Regina Justo Giugliani, 2008; Susiloretni et al., 2015) and complementary feeding outcomes (Bazzano et al., 2017). Yet many research studies have shown that engaging fathers and grandmothers in interventions across different contexts can be effective in supporting breastfeeding (Britton et al., 2007; Maycock et al., 2013; Mueffelmann et al., 2015; Olayemi et al., 2007; Pisacane et al., 2005) and complementary feeding (Allotey et al., 2022; Bilal et al., 2016; Mukuria et al., 2016).

While the results are mixed, these studies are important for advancing our understanding of the impact that fathers and grandmothers can have on IYCF; however, gaps in the evidence remain. Most studies have focused only on breastfeeding (Martin et al., 2020). Furthermore, few published studies conducted in LMICs have reported the social norms (‘collective awareness about the preferred, appropriate behaviours among a certain group of people’; Chung & Rimal, 2016) and attitudes and beliefs of these key influencers from their own points of view. Understanding these aspects from the influencers themselves is essential to develop and evaluate effective intervention and communication strategies to engage important child nutrition actors to improve IYCF and child health outcomes. The aim of this study is to complement existing evidence by exploring differences in attitudes, beliefs and social norms related to breastfeeding and complementary feeding from the perspective of mothers *as well as* fathers and grandmothers of young children and shifts in attitudes, beliefs and social norms over time in the context of a large‐scale IYCF intervention in Nigeria.

## 2 METHODS

### 2.1 Study overview and setting

This qualitative research was conducted within the context of an impact evaluation of the Alive & Thrive (A&T) intervention in Nigeria (Flax et al., 2022). As a global initiative, A&T contributes to better child nutrition by delivering high‐impact IYCF social and behaviour change interventions (including interpersonal communication, community mobilisation, advocacy and strategic use of data) at scale in LMICs. A&T is grounded in the socioecological model, which acknowledges the role that interpersonal relationships play in supporting optimal health behaviours, along with organisational, community and public policy factors (McLeroy et al., 1988). In this study, we focused our data collection on the individual and interpersonal levels of the socioecological model, where attitudes, behaviours and social norms were expected to be influenced by A&T's interpersonal communication and community mobilisation intervention components.

A&T's intervention is described in detail elsewhere (Flax et al., 2022). Briefly, in intervention communities, health workers were trained on IYCF practices, interpersonal communication skills and counselling techniques and data reporting; these health workers then delivered IYCF messages and materials to pregnant women, mothers of young children, and those who attended antenatal, postnatal and well‐ and sick‐child visits with them. Community volunteers (e.g., representatives from community organisations and community leaders) were trained and they conducted home visits and community meetings to discuss IYCF with pregnant women and families of young children. In both intervention and control communities, IYCF messages were delivered through mass media channels such as television, radio, posters, leaflets, billboards and vehicle ads. The theory of change for the intervention, which was tested by A&T in other countries (Sanghvi et al., 2013), hypothesises that interpersonal communication, mass media and community mobilisation, layered with advocacy and strategic use of data, will improve IYCF knowledge and practices among mothers and key influencers. The theory of change also hypothesises that these intervention components will also improve health worker capacity to deliver IYCF services and ultimately improve health outcomes (Sanghvi et al., 2016). The impact evaluation within which this qualitative research was nested sought to test the impact of the A&T intervention on early initiation of breastfeeding (EIBF), exclusive breastfeeding (EBF) and minimum dietary diversity (DD; Flax et al., 2022). This paper describes how those intervention components may have influenced the IYCF attitudes, beliefs and social norms of mothers of young children and key influencers over time.

In Nigeria, the prevalence of optimal breastfeeding and complementary feeding practices is low. Nationally, only 42% of children are breastfed within 1 h of birth, 29% are exclusively breastfed until 6 months of age, 23% of children of 6–23 months of age have an adequately diverse diet, and 42% have an appropriate meal frequency (NPC & ICF, 2019). A&T initially targeted Lagos and Kaduna States, in part because they represent very different social, ethnic and economic contexts and to use experiences related to IYCF intervention implementation from these states to scale the approach to neighbouring states. Lagos State includes Nigeria's largest city and is located in the South West zone. Kaduna State has urban areas but a largely rural population and is located in the North West zone. Women in Lagos tend to have higher levels of education than in Kaduna, and the percentage of poor households is lower in Lagos than in Kaduna (NPC & ICF, 2019). Higher education and wealth are associated with improved breastfeeding and complementary feeding practices in Nigeria (Ogbo, Agho, et al., 2015; Ogbo, Page, et al., 2015), and there are differences in IYCF indicators between the two states. For example, the prevalence of early breastfeeding initiation is 59% in Lagos and 36% in Kaduna, the prevalence of receiving a prelacteal feed is 24% in Lagos and 74% in Kaduna, and minimum DD is 41% in Lagos and 18% in Kaduna (NPC & ICF, 2019).

As part of the impact evaluation for the A&T initiative in Nigeria, we collected qualitative data at baseline to document social norms, attitudes and beliefs of mothers, fathers and grandmothers of young children related to IYCF to help inform the development of intervention communication strategies. We also collected qualitative data at the midline (mothers only) and endline from mothers, fathers and grandmothers to evaluate for changes in attitudes, beliefs and social norms related to IYCF during and after the intervention.

### 2.2 Study design and participants

We collected data in eight local government areas (LGAs)—the largest administrative sub‐units of states in Nigeria—assigned to receive the A&T intervention. Four LGAs were selected in Lagos, and four LGAs were selected in Kaduna. The LGAs in Lagos (Shomolu, Ajeromi Ifelodun, Kosofe and Surulere) are urban. In Kaduna, two of the LGAs are urban (Kaduna North and Zaria), while the other two are rural (Jaba and Kachia). At baseline, we conducted 16 focus group discussions (FGDs) with mothers, 16 FGDs with fathers and 16 FGDs with grandmothers, all with children or grandchildren less than 2 years of age. This structure was repeated at endline. At midline, we conducted 16 FGDs with mothers only. The number of FGDs for each type of participant was evenly divided across the two states, and FGDs were conducted with participants from the same LGAs. We determined the sample sizes based on the minimum needed to achieve saturation in qualitative research, the point at which additional data collection does not yield new information overall or within subgroups (Baker & Edwards, 2012; Guest et al., 2006). Baseline data collection took place in January and February 2017, midline data collection took place in June 2019 and endline data collection took place in January and February 2020.

Mothers were eligible if they were 15–17 years and married or 18–49 years because the Nigerian Constitution considers women <18 years who are married as adults. Mothers throughout the reproductive age range were included to allow us to capture data from older and younger mothers. Fathers were eligible if they were 18–50 years. This age range for fathers allowed us to include older and younger fathers without including men over 50, who may be more influential and affect participation by other focus group members. Grandmothers were eligible if they were at least 18 years. Other eligibility criteria for all three types of FGD participants included having a child or grandchild 0–23 months of age, no prior participation in other data collection activities for the impact evaluation and no relationship to another FGD participant.

Trained recruiters used a questionnaire to identify participants with medium or low socioeconomic status (SES) based on asset ownership (e.g., refrigerator, television, car and generator), household infrastructure characteristics (e.g., source of water and type of toilet) and level of education. Mothers and fathers were also recruited based on their age (15–29 or 30–49 years for mothers, 18–29 or 30–50 years for fathers). Groups comprised participants with similar SES and age (except for grandmothers) to prevent any influence by older respondents on younger respondents' responses. That is, younger mothers were in FGDs, separate from older mothers. Younger fathers were in FGDs, separate from older fathers. Grandmothers of all ages were in their own FGDs. In urban areas, recruiters identified buildings and households from which to recruit systematically. In rural areas, the recruitment team asked village leaders to help identify potential participants. Recruiters visited potential participants in their homes, used the screening form to check for eligibility, recruited and conducted informed consent procedures with the individual and informed them of the time and place for the FGD. Our target was eight participants per FGD.

### 2.3 Data collection

For each state, we employed a team of recruiters, moderators, notetakers and supervisors. Separate teams were needed because different languages are spoken in the two states (Hausa in Kaduna and Yoruba in Lagos). We selected moderators and notetakers based on their education, previous experience with qualitative data collection and fluency in the local language. Most of the team members participated in all three rounds of data collection. We conducted multiple‐day training for all team members for each data collection time point. The format and content of each training were the same. Following the training for all data collection time points, the data collection teams piloted the question guides in their respective states and modified translations as needed before starting data collection.

The FGDs were held in rented halls or empty community spaces and were digitally audio‐recorded. The FGD guides included questions on IYCF practices recommended and defined by the World Health Organization (WHO) (World Health Organization & Pan American Health Organization, 2003; World Health Organization, et al., 2008), three of which the A&T intervention focused on—EIBF, EBF and DD (specifically the consumption of green leafy vegetables [GLVs] and animal‐source foods [ASFs]). Other relevant topics, such as mothers working outside the home, alternate caregivers when mothers are away working and sources of advice on child feeding, were also included in the guides. The question guides at baseline used the first half hour after birth as the time frame for EIBF because this was the policy of the Nigerian Federal Ministry of Health at the time of data collection, although it differed from the WHO's recommendation to initiate breastfeeding within the first hour of birth. The policy was subsequently changed to align with the WHO guidelines, and the first hour after birth was used as the time frame for EIBF for midline and endline data collection. Questions related to knowledge asked respondents to describe what they knew about specific elements of IYCF (e.g., “What does the term exclusive breastfeeding mean to you?”). Questions related to attitudes, beliefs and social norms asked respondents to share what they and others thought about specific IYCF practices recommended by WHO and whether they agreed or disagreed with those recommendations (e.g., “What do you think about the advice to breastfeed a baby within 1 h of birth?”). We also asked about the facilitators, barriers, advantages and disadvantages of following these practices. The guides were developed in English and then translated into Hausa and Yoruba. Experienced transcriptionists prepared transcripts of the audio‐recorded FGDs, translating them directly into English. We reviewed the transcripts for completeness and clarity, and queries were verified against the recordings.

All procedures involving human subjects were approved by the institutional review boards at FHI 360 and RTI International and the ethics committees of the Kaduna State Ministry of Health and the Lagos State Government through the Lagos State University Teaching Hospital, Ikeja. We obtained written or thumb‐printed informed consent from all participants before collecting data, including permission to digitally record FGDs. Participants were given 1500 Naira (approximately 4 USD) after completing the FGD.

### 2.4 Data analysis

We used thematic content analysis methods to analyse the FGD transcripts (Gibbs, 2007). We created deductive codes based on the question guides for mothers and fathers/grandmothers and modified codebooks for midline and endline to reflect changes made to the question guides based on baseline results and evolving initiative needs. We also created inductive codes as needed throughout the coding process. The question guides for FGDs with fathers and grandmothers were very similar to each other; thus, their codes were the same. We used Dedoose (SocioCultural Consultants LLC) to code transcripts. The transcripts were divided among a team of analysts to code, and the team lead double‐coded 10% of each analyst's transcripts and provided feedback to ensure inter‐coder reliability. The team also met regularly to discuss coding questions and emerging themes. At baseline, we then developed data matrices for each participant group with one row for each FGD and columns for relevant codes and demographic variables (Miles & Huberman, 1994). The cells contained a summary of the findings from that FGD for each code and illustrative quotations. We used the matrices to sort the data and make comparisons between subgroups.

At midline and endline, we modified the data analysis process to better manage the volume of data collected and the larger analysis team. After coding was completed in Dedoose, we exported excerpts for each code into Excel, which then served as matrices for analysis. After analysing the matrices, we then created summary documents for each code to summarise the overall sentiments from the code, similarities and differences in findings between participant types and characteristics and illustrative quotes. We analysed data for each round of data collection separately and then compared the data across time points to detect changes in social norms related to IYCF, IYCF misperceptions and facilitators, barriers, advantages and disadvantages to implementing optimal IYCF practices, all according to each type of participant, state and age group.

## 3 RESULTS

### 3.1 Respondent characteristics

The number of participants and their characteristics are shown in Table 1. Mothers and fathers were, on average, in their early 30s, while grandmothers were in their mid‐50s. The average age of their youngest child or grandchild was approximately 11–12 months. Most participants were married. Mothers generally had similar levels of education across time points. Fathers at endline had slightly higher levels of education than fathers at baseline. Grandmothers at baseline had slightly higher levels of education than grandmothers at endline. Fathers had higher levels of education compared to mothers and grandmothers across time points. The majority of participants across time points were self‐employed, but about one‐quarter of mothers and grandmothers at baseline and endline reported being unemployed.

**Table 1:** Focus group discussion participant characteristics across all time points.

	Baseline			Midline	Endline		
	Mothers (*n* = 128)	Fathers (*n* = 130)	Grandmothers (*n* = 127)	Mothers (*n* = 141)	Mothers (*n* = 128)	Fathers (*n* = 128)	Grandmothers (*n* = 128)
Mean ± SD							
Mean age, years^a^	30.3 ± 6.1	31.8 ± 7.1	56.8 ± 10.6	29.3 ± 5.5	30.1 ± 5.9	31.9 ± 6.9	56.7 ± 9.0
Mean age of youngest child, months	11.9 ± 6.0	11.8 ± 6.7	11.7 ± 6.7	11.2 ± 6.4	11.3 ± 5.9	11.4 ± 5.9	10.8 ± 5.7
Mean number of assets (out of 20)	6.6 ± 3.1	6.5 ± 2.8	6.1 ± 3.9	7.1 ± 3.5	7.2 ± 3.7	7.6 ± 3.5	7.8 ± 4.2
*n* (%)							
Married	125 (97.7)	126 (96.9)	114 (89.8)	141 (100.0)	124 (96.9)	127 (99.2)	100 (78.1)
Level of education^b^							
Primary school, any amount	18 (14)	19 (14.6)	60 (46.5)	18 (12.8)	16 (12.5)	5 (3.9)	44 (34.3)
Secondary school, any amount	69 (53.91)	58 (44.6)	36 (27.9)	70 (49.6)	67 (52.3)	54 (42.2)	53 (41.4)
University or polytechnic school, any amount	40 (31.3)	44 (33.9)	29 (22.5)	34 (24.1)	30 (23.4)	42 (32.8)	12 (9.4)
Post‐university, any amount	0 (0)	8 (6.2)	1 (0.8)	14 (9.9)	12 (9.4)	24 (18.8)	6 (4.7)
Employment^c^							
Employed full time	8 (6.3)	22 (16.9)	9 (7.1)	6 (4.3)	10 (7.8)	20 (15.6)	5 (3.9)
Employed part time	10 (7.8)	18 (13.8)	15 (11.8)	5 (3.5)	2 (1.6)	7 (5.5)	2 (1.6)
Self‐employed	76 (59.4)	80 (61.5)	65 (51.2)	107 (75.9)	85 (66.4)	98 (76.6)	86 (67.2)
Unemployed	29 (22.7)	3 (2.3)	32 (25.2)	23 (16.3)	31 (24.2)	3 (2.3)	35 (27.3)

^a^ Data missing for 1 mother at baseline; 4 mothers at midline.

^b^ Data missing for 1 mother, 1 father and 1 grandmother at baseline; 3 mothers at midline; 1 mother and 1 grandmother at endline.

^c^ Data missing for 5 mothers, 7 fathers and 6 grandmothers at baseline.

### 3.2 Breastfeeding

#### 3.2.1 Early initiation of breastfeeding and colostrum

We identified six themes related to EIBF; these can be found in Table 2, along with illustrative quotes. At baseline, most participants, and especially mothers, agreed that the advice to breastfeed a baby within the first half hour of birth is good (Theme 1). Almost all mothers at baseline and midline also agreed with the advice to feed babies colostrum. By endline, these sentiments increased among fathers and grandmothers. Across all time points, those who agreed with the practice of EIBF and feeding colostrum said they did so mainly because of the perceived advantages (Theme 2). At both baseline and endline, participants described those advantages as follows: the first breast milk makes the baby strong and is good for growth; it is good for the baby and makes the baby healthy; it is good for the baby's brain, and it contributes to baby's immune system and prevents or fights illnesses. Mothers and grandmothers across time points also noted that colostrum helps flush the baby's system and facilitates defecation.

**Table 2:** Key themes related to early initiation of breastfeeding and colostrum and illustrative quotes.

Themes related to early initiation of breastfeeding and colostrum	Illustrative quotes
1. Across time points, most mothers believed feeding the baby within ½ hour to 1 h of birth was good. These sentiments increased among fathers and grandmothers by endline.	*[Breastfeeding the baby within the first half hour of birth is good] because it is for the newborn child. The child's first food must be what came out of the mother. The child must have been hungry, though he or she has been eating in the belly, but you can't compare that with the outside. So it's good to breastfeed the child first*.—Older mother from Lagos, baseline
	*The breast milk coming out from the mother's breast is good, that is why it is good to feed the baby with it within 30 min after giving birth*.—Younger mother from Kaduna, baseline
	*In the past, you know, they didn't use to give them this yellowish breast milk. We used to drain it and throw it away. But now we know how important it is, and we also understand the benefits directly for every child that takes it*.—Grandmother from Kaduna, endline
2. Across time points, participants’ support for EIBF was mostly attributed to its perceived advantages.	*For me, I think it's good for us to give our children breast milk within the first 30 min because when we give the baby breast milk, it will make the baby strong and healthy*.—Older mother from Lagos, baseline
	*Me, I noticed that when I give any of my children the first breast milk, they will toilet very well. The first breast milk will help in flushing their system very well*.—Younger mother from Lagos, baseline
	*The doctor has said it makes the child healthy, if the child is given breast milk at the time of birth. It also protects the child from illnesses that affect children. If the child sucks his or her mum's breast within that time, God‐willing, the child would be protected against illness. That is why we are determined to give them breast milk in that time*.—Younger mother from Kaduna, midline
	*[Colostrum] is very useful for the child. It improves the child's health. If the child starts to defecate, it seems as if it is washing the child's stomach. He will start defecating with the black colour, then later he properly defecates. It seems it flushes out dirt from the child's stomach*.—Grandmother from Kaduna, midline
	*Breastfeeding the child within 1 h will give the child energy and, like he said, the baby's brain will be well developed, and he will be intelligent*.—Younger father from Kaduna, endline
	*It improves eyesight, and if there is anything that is disturbing the child in his stomach, it will flush it out, and the child will feel okay because that breast milk is like a medicine to him. It improves strength and health*.—Younger mother from Kaduna, endline
3. The most common facilitators for EIBF are having a safe and healthy delivery (baseline), being well‐nourished during pregnancy and after delivery (all time points), support from family and health workers (all time points) and antenatal care and education (all time points).	*If your wife delivered safely, there is no excuse she can give for not breastfeeding the child [within 1 h of birth]*.—Younger father from Lagos, baseline
	*If the woman ate a balanced diet when she was pregnant, then she can breastfeed her child within that 30 min of birth*.—Younger mother from Lagos, baseline
	*The mother should eat well within that one hour so she can be able to breastfeed the child. She should take hot tea, pap (thick porridge). She should take hot amala [thick pudding made from dried yam powder] with ewedu [green leafy vegetable used to make a soup] so she would lactate well*.—Older mother from Lagos, midline
	*If the mother has someone that can assist her, because after giving birth you don't have enough strength. So if you have someone that assists at that moment, it will make the [first] breast milk more easily available*.—Younger mother from Lagos, midline
	*As a husband, you allow your wife to go to her antenatal check‐up appointments because that is where she will be informed [about breastfeeding within 1 h of birth]*.—Younger father from Kaduna, endline
4. Some participants across time points had reservations about EIBF and colostrum. At endline, fathers were still the most likely of all participants to disagree with the advice to feed babies colostrum.	*When a woman gives birth, at first, the breast milk might not be good. It is supposed to be squeezed and thrown away*.—Older father from Kaduna, baseline
	*Sometimes when a mother gives birth, there is this breast milk that used to come out. The breast milk is not good. You'll see the appearance of it, and it looks yellow‐yellow. When such things happen, we will fetch some leaves of pawpaw and soak it, and then wash the breast and get rid of the yellowish substance of milk until the breast milk coming out is pure white. That is when the mother will breastfeed the child. If we let the child feed on that yellow substance of milk, maybe the child might be ill, because it is not good*.—Grandmother from Kaduna, baseline
	*I believe the first breast milk should be expressed out a little because it's like water that is stored in a tank for long. If you did not open the tank for a week, by the time you want to open it, the mouth would have been rusted. If you did not flush the rusted part away, it can cause harm into the body*.—Younger father from Lagos, endline
5. Across time points, participants acknowledged water is commonly given right after birth for a variety of reasons.	*In this community, this is one of the inherited traditions, and that is what our religion asked us to do. For the honey, when given to the child with warm water, it washes the child's stomach to enable him to drink the breast milk* —Younger father from Kaduna, baseline
	*Some will give water because some children, immediately, when [a mother] gives birth to them, they will start crying. So they will give water*.—Grandmother from Lagos, baseline
	*Some mothers might be very weak and won't be able to attend to the child, so they use the water to support the baby*.—Younger mother from Kaduna, midline
	*Some mothers’ breast don't lactate easily, and such baby will be given warm water first and mix with glucose*.—Grandmother from Lagos, endline
	*They believe in what they believe, because they, too, consider how thirsty a child could get. They gave us water when we were babies, though not as hygienic*.—Younger mother from Kaduna, endline
6. Across time points, counselling from health workers during antenatal care was considered an important source of information and facilitator of EIBF and use of colostrum.	*I agree [with the advice to breastfeed within 30 min of birth] because when I gave birth to my baby, the nurse told me that there is a yellow milk that comes first, that it's very important for the baby*.—Younger mother from Lagos, baseline
	*I don't know [colostrum's] relevance … It's the women who know. If I know, I'll let them feed the baby with it. I will agree with doctors’ advice on feeding the child with it as the first meal for the baby*.—Younger father from Kaduna, baseline
	*I think it's a good advice [to breastfeed within 1 h of birth] because I know that if it's not good, they [health workers] won't tell you to give your baby breast milk. So I know it's hygienic, and that's why they tell us to breastfeed*.—Older mother from Lagos, midline
	*Yes, we all agree to it [the advice to breastfeed within 1 h of birth] because we were advised by doctors to breastfeed immediately after giving birth*.—Older mother from Kaduna, midline
	*Because the medical professional said that we should feed the child breast milk within 1 h, the midwife should try and give the mother her child to feed him*.—Younger father from Kaduna, endline
	*Also, I've heard it over and over on the radio and in conversations with doctors and health workers on the benefits of breastfeeding a child that yellowish breast milk in the first few days. We have tested it, and we have seen that the benefits are true*.—Grandmother from Kaduna, endline

Participants noted several main facilitators of EIBF and feeding colostrum (Theme 3). At baseline, the most important facilitator mentioned by all types of participants was having a safe and healthy delivery because the mother would be in a better physical condition to initiate breastfeeding. Participants across all time points also said it is important for mothers to be well‐nourished during pregnancy and right after delivery to facilitate early breastfeeding because eating well and drinking fluids support milk production. Receiving support from family members was also mentioned as a facilitator of EIBF and feeding colostrum across all time points. Older mothers in Kaduna at midline and grandmothers at endline also believe mothers should prepare and clean their breasts or nipples during pregnancy and before delivery to facilitate breastfeeding within the first hour after birth.

However, some participants across time points had reservations about EIBF and colostrum (Theme 4). Participants in several grandmothers' and fathers' FGDs at baseline said the advice to breastfeed within a half hour of birth is only good if the mother is “in good condition” after delivery and if the baby is strong enough to breastfeed. Some fathers at baseline also felt water should still be given before or in addition to breast milk during the first half hour or that the first breast milk should be discarded and not given because it is dirty. The sentiment that colostrum is dirty (indicated by its colour and perceived length of time in the breast during pregnancy) and therefore should not be given to babies was most prevalent among fathers and grandmothers across time points compared to mothers. At baseline and endline, fathers had limited knowledge of colostrum, and at endline, they were still the most likely of all participants to disagree with the advice to feed babies colostrum. Mothers and grandmothers at baseline also acknowledged traditional beliefs may still keep some mothers from giving colostrum to their children. However, they also articulated that mothers used to throw away the first breast milk, but the awareness of its importance was increasing, so it was less frequently discarded.

Across all time points, participants also acknowledged that water is commonly given right after birth (Theme 5). The primary reasons for doing so included: the baby is thirsty after delivery, water is important and good for the baby, it helps to “open up” their digestive systems for breast milk, it clears the baby's eyes, that it is part of cultural birth rituals and holy water is given for religious reasons. Various barriers to EIBF were also cited by participants as reasons for not practising EIBF, the most common being the mother may not have the strength or maybe in too much pain right after delivery (all time points); the mother's milk may not yet be flowing (all time points); the mother had a Caesarean section (all time points) and is unconscious after delivery or needs time to recover; the mother may have been counselled not to breastfeed if she is sick, including with HIV (baseline and midline), or the mother is sick (e.g., HIV, tuberculosis and hepatitis, breast cancer) and cannot begin breastfeeding until she receives treatment (endline).

Across time points, health workers and antenatal care were seen as important sources of information and facilitators for EIBF (Theme 6). Those who agreed with EIBF and feeding colostrum across time points cited recommendations from health workers as a main reason for supporting the practice, along with health advantages. Participants across all time points said that attending antenatal care visits can facilitate EIBF and feeding colostrum through increased awareness and ensuring the mother's health before delivery. At endline, many participants noted a lack of knowledge about breastfeeding, including due to lack of attendance at antenatal visits, which could be a barrier to EIBF. Many fathers at baseline indicated that they did not know a lot about colostrum and thus did not know if the advice to feed it to babies was good or bad, but they said they were willing to learn and would accept the advice to feed it to babies if a health professional recommended it to them.

#### 3.2.2 Exclusive breastfeeding

We identified five themes related to EBF; these can be found in Table 3, along with illustrative quotes. Across all time points, most mothers agreed the advice to exclusively breastfeed a baby is good; this sentiment increased among fathers and grandmothers by endline, but support for the practice was more prevalent among fathers than grandmothers (Theme 7).

**Table 3:** Key themes related to exclusive breastfeeding and illustrative quotes.

Themes related to exclusive breastfeeding	Illustrative quotes
7. Across time points, most mothers agreed exclusive breastfeeding was good. This sentiment increased among fathers and grandmothers by endline.	*Yes, [exclusive breastfeeding] is useful because there is food in it, and it helps the child*.—Older mother from Kaduna, baseline
	*[I say exclusive breastfeeding is good] because it's useful. It builds them up. I am also doing the exclusive breastfeeding*.—Older mother from Kaduna, baseline
	*[Exclusive breastfeeding] is the best*.—Older mother from Lagos, midline
	*We are in agreement [that exclusive breastfeeding is good]. In the past, we thought that giving other things such as gripe water is also exclusive, but now we know*.—Older mother from Kaduna, midline
	*It is good that the child should be breastfed for that 6 months as advised so that the strength of the child and the health of the child will be optimum*.—Younger father from Kaduna, endline
8. Across time points, participants cited numerous benefits of EBF, which were the main reasons participants supported EBF.	*The child gains health‐wise, and the breastfeeding mother is healthier*.—Older mother from Kaduna, baseline
	*Feeding a child with breast milk from birth up to 6 months, I know it adds more energy to the child. It makes him healthier, and there are other things he gets that strengthen his immunity from that breast milk. The milk contains everything because you will see even the wisdom he exhibits is different from the other babies that don't feed on the breast milk, but other food*.—Older father from Kaduna, baseline
	*Once you do exclusive breastfeeding, your baby would be strong and healthy, that you would hardly spend much on the child's health care. Nothing would happen to the child*.—Younger mother from Kaduna, midline
	*We are told that when we breastfeed our babies well, the child would show us mercy when we are old, but if we did not breastfeed our babies, they would not show us mercy. That is what Yoruba people usually say*.—Older mother from Lagos, midline
	*Honestly, I agree with the advice because I see this type of breastfeeding as quite good for babies. If you look at the babies that are exclusively fed, you see them stronger, and you see less amount of diseases or sickness in the child. It's quite helpful*.—Grandmother from Kaduna, endline
	*[Exclusive breastfeeding] is a good advice because if you breastfeed a child for 6 months, there would be a difference between that child and a child who takes water. The child's brain would be sharp, the child would think well, and the child would have strong bones. When such child talks, it would be audible. I tried it for my children and my grandchild, and it works*.—Grandmother from Lagos, endline
9. Participants cited the mother eating enough and eating well so that she can produce enough breast milk and have the strength to breastfeed as the main facilitator to EBF across time points.	*It is good for the mother to eat food and be satisfied so that the breast milk will come to her. The baby will have enough to take to be satisfied*.—Younger mother from Kaduna, baseline
	*When she takes enough vitamins and good food, [she can exclusively breastfeed]*.—Older father from Lagos, baseline
	*If there is not enough food in the house, I will search for food and prepare it for them. The better she is fed, the more milk she will produce*.—Grandmother from Kaduna, baseline
	*If a mother eats well, she will be able to [exclusively] breastfeed. If the mother doesn't eat properly, then breast milk won't be enough for the baby*.—Younger mother from Lagos, midline
	*The mother should be eating good food so that she will have enough breast milk [for exclusive breastfeeding]*.—Older mother from Kaduna, midline
	*If the husband is stable financially and can provide for the needs of the mother and the child. If the mother is at peace, and there is enough food at home, it will make it easier [for her to exclusively breastfeed]*.—Grandmother from Lagos, endline
10. Participants described a strong belief in the importance of feeding the baby water (more so among fathers and grandmothers), which played a role in hindering the acceptance and practice of EBF among some participants.	*You shouldn't be breastfeeding a child just like that [exclusively for 6 months]. There must be time for water and breastfeeding because water plays its own role, while breastfeeding plays its own role, too*.—Grandmother from Lagos, baseline
	*It's punishment, because it's only a wicked mother that will do that [breastfeed exclusively]. As an adult, if I don't have something to eat or drink, I will not have the strength to do anything. So, for the mother not to give the baby semisolid food or baby food or water, that's wickedness*.—Younger mother from Lagos, baseline
	*I personally understand that it is unfair to keep a child without giving him water to drink or other food because it is not possible for him to be satisfied with only breast milk, and then he will disturb his mother*.—Younger father from Kaduna, baseline
	*A child needs water. Because nobody can eat without water. If you eat without water, you wouldn't feel comfortable*.—Older mother from Lagos, midline
	*Some will just think that it is your own opinion [about exclusive breastfeeding] because of the school you went to, that you will kill the child with thirst. And if you are not vigilant, some of them will steal the child and give water. We usually have most of these issues*.—Older mother from Kaduna, endline
	*The advice [to exclusively breastfeed] is good, but we still need to give water so that the child would be strong*.—Grandmother from Lagos, endline
	*Giving water and breast milk is very good. Water is very good for the system. Not only babies, even for adults, too. It would give them more strength*.—Younger father from Lagos, endline
11. Health workers were considered an important source of information and facilitator for EBF across time points.	*Breastfeeding is said to be important. Those who did the research told us to do so. What they found is that we should be breastfeeding children*.—Older mother from Lagos, baseline
	*The doctor said we shouldn't be giving them water, that we should be breastfeeding them. That [breast milk] has what it is doing in the body. After 6 months, we can start giving them other things*.—Grandmother from Lagos, baseline
	*When I heard from the health workers that breast milk is the only right food for the baby, I decided to stick to it. Even when I had pressure from people to give the baby water, I only responded with, ‘Okay’, but I shun their advice by continuing with the exclusive breastfeeding*.—Younger mother from Kaduna, midline
	*Breast milk is the best for babies. But during our days, we used water. But when my child gave birth, in the morning, I would give the child water, then the mother would breastfeed all through the day. But since it's an expert advice not to give water, then we won't be giving water again*.—Grandmother from Lagos, endline
	*As a husband, you allow your wife to go to her antenatal check‐up appointments because that is where she will be informed [about breastfeeding]*.—Younger father from Kaduna, endline

Across all time points, participants cited numerous benefits of EBF (Theme 8). Most of the benefits are primarily related to child health and development, such as preventing sickness, helping children grow and develop physically and mentally and making them strong. Mothers and fathers at baseline also described the bond that is developed between mothers and children through breastfeeding. Mothers at baseline and midline also said it helps them as mothers to be healthier. At midline, mothers also cited advantages around family planning, greater economic security because health care costs are reduced due to the baby's improved health and the child's appreciation of being breastfed when he/she is older.

All types of participants across time points cited the mother eating enough and eating well so that she can produce enough breast milk and have the strength to breastfeed as the main facilitator of EBF (Theme 9). At midline, some mothers also noted that women need to have the economic security to eat well and indicated a role for government to support this.

Participants reported a strong belief in the importance of feeding the baby water, either as an individual belief (more so among fathers and grandmothers) or as a cultural belief, and this belief played a role in hindering the acceptance and practice of EBF among some participants (Theme 10). At baseline, grandmothers and fathers were more likely than mothers to disagree with the advice to exclusively breastfeed, mainly because of the belief that water should also be given. They said (along with some mothers) that water should be given to babies because it quenches the infant's thirst, is necessary for life and cleanses the stomach to facilitate defecation. Some mothers at midline in both Lagos and Kaduna indicated that EBF could contribute to poor child health because of the importance of water in a child's diet.

Some fathers and grandmothers at baseline also noted that EBF is not always practical (e.g., EBF keeps mothers at home too much or the challenges of feeding the baby when the mother is not available). Other barriers to EBF cited by all types of participants across all time points included the following: mothers may not produce enough milk, believing the child is no longer satisfied by breast milk alone, mothers being ill and/or lacking strength to continue breastfeeding, mothers' lack of time or returning to work and having limited maternity leave, mothers not wanting to lose their shape and/or have their breasts to sag, mothers just deciding to stop and a lack of knowledge about the importance of EBF.

Health workers were seen as important sources of information and facilitators for EBF across time points (Theme 11). Participants credited health workers as the source of their knowledge of EBF benefits, which played a role in mothers' acceptance of the practice. Mothers at midline also emphasised the role of health education among mothers, other family members and the community at large as facilitators for EBF. Most participants at endline (more so among fathers, younger parents and those from Kaduna) also noted receiving information about the importance of EBF through antenatal care as a facilitator.

### 3.3 Complementary feeding—DD

We identified four themes related to DD; these can be found in Table 4, along with illustrative quotes. Participants' attitudes and beliefs regarding DD were consistent across types of participants. Participants across all time points recognised the importance of DD offering young children a variety of foods (Theme 12). Although a specific number of recommended food groups was not mentioned, all types of participants across time points described how children should eat a variety of foods each day and characterised DD as changing a child's food throughout the day to ensure he or she is eating a varied or “balanced” diet, which has “all the classes of food” and nutrients. Many participants from each category specifically noted that protein and carbohydrates as important for children. Fathers at baseline also mentioned fruits, vegetables and vitamins.

**Table 4:** Key themes on dietary diversity and illustrative quotes.

Themes related to dietary diversity	Illustrative quotes
12. Participants across all time points recognised the importance of offering young children a variety of foods.	*For the mother, she can eat the same food today, tomorrow and the next day, until the end of the week. But for the child, it is not like that. If the child ate tuwo [stiff porridge derived from grain (e.g., maize, rice, guinea corn)] today, tomorrow you'll like to change it for the child. Some when they eat in the morning, it is not the same they will eat in the evening. They will change meals*.—Older mother from Kaduna, baseline
	*What we should learn first is to know how to balance food for the children. Like, the protein won't be too much, and it won't lack carbohydrate. In fact, you should teach the children all types of foods, even a child of 23 months that doesn't repeat the garri [thick pudding made from cassava flakes] for three square meals. We just make sure the foods are balanced diets*.—Younger father from Lagos, baseline
	*And also, the way we give [food] to them, that is very important. We should be changing the foods*—*like protein in the morning and something else in the afternoon. We should give them what would give them strength in them morning and what would give them energy in the afternoon and what would give them blood in the evening. We shouldn't stick to one type of food for the whole day. It is not good to do that It is harmful for the child*.—Younger mother from Kaduna, midline
	*Because if a child eats, the food becomes very, very good for the child's growth and development, especially when it is balanced. If you give a child too much carbohydrate, it develops a protruding tummy. But if you balance it with proteinous foods, he grows better*.—Younger father from Kaduna, endline
13. Participants across all time points recognised specific health benefits of green leafy vegetables and animal‐source foods.	*It increases their strength and makes them more agile at all times. Because the thing [green leafy vegetables] contain medicinal substances that fight some diseases. When you eat these things, God willing, you will not contract any disease*.—Older father from Kaduna, baseline
	*[Animal source foods] build their body. They will grow in sound health. It will make your child to have good upbringing, brainy in school, and to have strength and vigour. When they play, they jump all over the place*.—Younger mother from Kaduna, baseline
	*[Green leafy vegetables] will supply blood…it will build their bones and their immune system*.—Older mother from Lagos, midline
	*[Egg, fish]…they are proteinous food. It would make the baby grow well and also fight disease in the child's body*.—Grandmother from Lagos, endline
	*They all have advantage. Like ugwu [jute leaves], it supplies blood and when you give a child dark green leafy vegetable, they would be looking fresh*.—Younger father from Lagos, endline
14. Across time points, participants noted some disadvantages and barriers to green leafy vegetables and meat that keep parents from feeding them to their children, specifically related to causing diarrhoea and difficulty with chewing and swallowing; although by endline, most participants’ perceptions of disadvantages had decreased, provided that these foods are consumed in moderation.	We all know that vegetables are good for the body. If there are other ways to prevent children from purging after eating them, then every parent is ready to be feeding them vegetables.—Older mother from Lagos, baseline
	Their stomach is not yet strong. Fish is the best for them because they can't chew meat.—Grandmother from Lagos, baseline
	*Some of the grandparents if they [give the child meat], people will say it can spoil the child and can make the child to steal. That is what we fear about it*.—Grandmother from rural Kaduna, baseline
	*When you give them without washing [vegetables] with salt, or when you cook it in the wrong way, and the way you feed the child, too, may lead to sickness, like diarrhoea*.—Younger mother from Kaduna, midline
	*Meat is good, but it must not be too much. It can give them worms*.—Older mother from Lagos, midline
	*There are some green vegetables that you can give children, and they start purging*.—Younger father from Kaduna, endline
	*Also, it won't digest because they don't have teeth to chew the meat*.—Older mother from Lagos, endline
	*There are no disadvantages, but too much of everything is not good*.—Grandmother from Kaduna, endline
15. Participants highlighted several facilitators for feeding children a variety of foods. Money was the most prominent facilitator, followed by preparation techniques that can facilitate children chewing green leafy vegetables and animal‐source foods.	*You know, it involves money, if you say you want to give the child different foods. There are certain foods that you would want to prepare for your child…I will prepare it for him, if I have the means to do that. But if I don't have the means, whatever I eat is what I will give him to eat*.—Grandmother for Kaduna, baseline
	*The real thing is financial. The family may not be financially buoyant. Because these foods we are talking about cost money. So for you to take care of your children and baby is money*.—Older father from Lagos, baseline
	*Like for a child, you will need ewedu [a green leafy vegetable] that you have ground, and it is already in small bits. That will make it easy to digest. Because at times, the vegetable is too big, and the child's digestive system can't carry such*.—Older father from Lagos, baseline
	*When there is no money, you won't be able to get varieties of food for the child*.—Older mother from Lagos, midline
	*When the parents are employed, have money, are well‐paid, are employed and all that [is what helps parents feed children a variety of foods]*.— Grandmother from Kaduna, endline
	*Because if there is no money, no work, no means of getting [variety of foods], then there is a problem. It becomes difficult to feed the child*.—Older father from Kaduna, endline
	*We should cook [green leafy vegetables] until they're tender, so that they can eat it. We would cut it into tiny bits and cook it until it's soft*.—Grandmother from Lagos, endline
	*Moderator: What would make it easier [to give children animal source foods]?*
	*Mother 1: We cook it and make it soft*.
	*Mother 2: For fish, we debone it and mash it very well*.—Older mothers from Lagos, endline

All types of participants across time points recognised specific benefits of GLVs and ASFs (Theme 13). Participants said that GLVs help children be healthy, grow well, have strength, be agile and ‘fresh’ (meaning the child looks well taken care of) and increases a child's blood volume, though at endline, this was more common among fathers and grandmothers than mothers. At baseline and endline, some mothers and fathers articulated that GLVs have ‘medicinal’ properties. At endline, many participants (though more so among grandmothers) also specifically noted GLVs can help fight infection and prevent illness because they contain vitamins and are good for wound healing. In general, across time points, all types of participants said ASFs enhance children's growth, development and health, notably because of the protein and vitamins they contain. Mothers and fathers in Kaduna at baseline also noted that ASFs can ‘increase a child's blood’, similar to GLVs. At endline, this sentiment was more common among fathers and grandmothers. At baseline and endline, all types of participants also said ASFs enhance cognitive development and eyesight.

Participants did mention some disadvantages and barriers to GLVs and meat that keep parents from feeding them to their children (Theme 14). Across time points, participants highlighted how GLVs cause ‘purging’ (i.e., diarrhoea) in young children; many participants noted diarrhoea and sickness occur more frequently if GLVs are consumed in excess or if they are not washed or prepared appropriately. Mothers and grandmothers in Lagos at baseline suggested that the only way to avoid purging was to wait until children get older to feed them GLVs so their systems can handle it. Most GLVs were also thought to be too difficult for young children to chew and swallow. The only exception is ewedu, which is used to make a ‘slippery’ soup. Participants from Lagos at baseline and mothers at midline also said meat is too difficult for children to chew and digest and could cause worms if given in excess, whereas fish and eggs are easy to chew and digest and are more affordable than meat. Mothers from Kaduna at baseline and mothers at midline said meat spoils children and makes them ‘steal’ (take food from others or from the household's cooking pot without permission) and ‘beg’ (ask for food from nonfamily members when outside the household). All participant categories at baseline also said children rejecting new foods or being selective prohibits them from eating a variety. All types of participants at baseline and endline (more so among grandmothers) and mothers from Lagos at midline said that laziness, carelessness and lack of time on the part of the mother or parents was a barrier to feeding children a variety of foods. By endline, most of all types of participants (although more so among those from Kaduna) said that there were no disadvantages to feeding children a variety of foods.

Participants highlighted several facilitators for feeding children a variety of foods (Theme 15). All types of participants across all time points cited money or lack thereof as the most important facilitator or barrier to feeding children a variety of foods. Other facilitators noted at baseline and endline included cutting GLVs and meat into very small pieces and cooking them well so they become very soft. Participants from Lagos at baseline and endline also noted that pre‐chewing meat or grinding or blending it would make it easier for children to chew and swallow.

## 4 DISCUSSION

We explored the attitudes, beliefs and social norms related to IYCF of mothers and key influencers of child feeding in two Nigerian states. Overall, we found more differences in attitudes, beliefs and social norms for EIBF and EBF across participant types than for DD. Although most participants (especially mothers) agreed that EIBF and EBF are good and offer health benefits, fathers and grandmothers had more reservations about the advice, particularly because of the belief that colostrum is dirty and water should be given to babies. Fathers knew the least about colostrum across time points and, at endline, were the least likely of all participants to agree with the advice to feed babies colostrum. However, by endline, there appeared to be a shift towards acceptance of EIBF and EBF among fathers and grandmothers. We did find a few similarities in results for EIBF and EBF, including the importance of mothers eating enough and eating well to support breastfeeding and the misconception that babies should be given water. Furthermore, all types of participants acknowledged the nutritional and health benefits of GLVs and ASFs but described how GLVs may cause diarrhoea, and they may be too difficult for children to chew and digest, among other barriers. Across time points, all types of participants highlighted the importance of health workers as important sources of IYCF knowledge and facilitators to following recommended practices.

Evidence on the influence and impact of fathers and grandmothers on child feeding has increased in recent years, and their inclusion in IYCF interventions and programmes is supported by the socioecological model, among other theories and models of behaviour change. However, most studies explore IYCF attitudes and beliefs from the mother's perspective or from quantitative data about behaviours rather than collect data from the key influencers themselves. To our knowledge, no other published papers have reported on mothers', fathers' *and* grandmothers' perspectives on IYCF over time within the context of an intervention. A recent systematic review (Earle & Hadley, 2018) identified only 20 qualitative studies that captured men's views and experiences related to IYCF. These studies represented 457 men and were published between 2000 and 2016; most of the studies in the systematic review only focused on breastfeeding, and only one study was conducted in a low‐income country (Uganda) (Engebretsen et al., 2010). The study in Uganda, along with other work in Cameroon (Reinsma et al., 2012), was similar to our study in that it explored the views of both mothers and influencers. These studies had similar results as ours regarding views on breastfeeding, along with social pressures contributing to the mother using suboptimal practices. Qualitative studies in Mozambique (Arts et al., 2011) and Zambia (Fjeld et al., 2008) found discrepancies in perceptions of EBF among mothers, fathers and grandmothers and a strong influence from grandmothers over a woman's IYCF practices, although respondents wary of EBF were willing to learn more from health workers, similar to fathers in our study related to colostrum. Our results also align with the findings of systematic reviews from Bazzano et al. (2017) and Balogun et al. (2015), including the belief that colostrum is not good for babies and that it is discarded until the white milk comes in; similar facilitators and barriers to EBF and barriers to feeding children a variety of foods were also found in those studies and in ours.

Moreover, many studies on the influence and impact of fathers and grandmothers on IYCF globally focus on identifying associations between influencer involvement and child‐feeding practices or anthropometric outcomes (Emmott & Mace, 2015; Hunter & Cattelona, 2014; Mueffelmann et al., 2015; Olayemi et al., 2007; Rempel & Rempel, 2004; Rempel et al., 2017; Susiloretni et al., 2015) or were evaluations of interventions that included influencers (Bich et al., 2015; Molzan Turan et al., 2001; Mukuria et al., 2016; Nguyen et al., 2018; Rosane Odeh Susin & Regina Justo Giugliani, 2008; Tadesse et al., 2018; Tokhi et al., 2018) rather than formative research dedicated to understanding influencers' ICYF knowledge, attitudes and beliefs. Conducting formative research *before* developing an intervention is essential to design a more effective intervention (Bentley et al., 2014; Paul et al., 2011). Many interventions and large‐scale programmes—including the A&T initiative in Nigeria—conduct formative research to inform intervention and programme design and communication strategies, such as to determine which IYCF practices should be prioritised for promotion and how messaging for those practices should be tailored; secondary audiences to target, in addition to mothers of young children; the training needs of healthcare providers who would deliver messages; and which communication channels are accessible to and trusted by potential programme beneficiaries (Baker et al., 2013; Sanghvi et al., 2013; Thuita et al., 2015). Reports with formative research findings, however, appear more prominently in the grey rather than peer‐reviewed literature, limiting their accessibility and impact beyond the intervention or programme for which they were intended. Moreover, many projects face time and budgetary constraints, potentially limiting the ability to conduct qualitative research before an intervention or programme. Tools such as the Focused Ethnographic Study (Pelto & Armar–Klemesu, 2014) and the Rapid Ethnographic Assessment (Sangaramoorthy & Kroeger, 2020) are helpful in these scenarios.

In addition to capturing the perspectives of fathers and grandmothers of young children as well as of mothers all within the same study and publishing results in a peer‐reviewed publication rather than grey literature, this study is also unique in that we collected qualitative data at multiple time points of an intervention, particularly due to data collection from mothers at midline. Although many results were static from baseline to endline with the exception of some nuances, there did appear to be a shift in acceptance of EIBF and EBF among fathers and grandmothers by endline. There are several potential explanations for this. First, because mothers of young children are usually the target population for IYCF interventions, fathers and grandmothers may have had more potential to respond to IYCF messaging and change their perspectives on issues than mothers. Additionally, there were more discrepancies in opinions and knowledge of breastfeeding than DD across types of participants, leaving more room for convergence of perspectives regarding breastfeeding throughout the intervention.

It is important to understand prevalent social norms related to IYCF and to delineate fathers' and grandmothers' beliefs about IYCF because of the potential impact on child feeding (Balogun et al., 2015; Bazzano et al., 2017; Martin et al., 2020; Martorell & Zongrone, 2012; Negin et al., 2016; Otoo et al., 2009; Tamiru et al., 2013). Knowledge, attitudes and beliefs may inform the type and impact of support that someone offers (Rempel & Rempel, 2004). Furthermore, different types of support likely have different pathways to influencing behaviours and both positive and negative outcomes (Emmott & Mace, 2015), so understanding the knowledge, attitudes and beliefs *in addition to* roles in a specific context is an essential condition for designing effective interventions. Although the relationship between beliefs, roles and actual feeding practices was beyond the scope of our qualitative research, evidence from other studies suggests that knowledge, attitudes, beliefs and perceived support from family members can improve concurrently with actual improvements in IYCF practices (Arusei et al., 2011; Bich & Cuong, 2017; Bootsri & Taneepanichskul, 2017; Flax et al., 2022; Nabulsi, 2011; Rempel et al., 2020; Shao Mlay et al., 2004; Su & Ouyang, 2016; Tamiru et al., 2013; Ulak et al., 2012). These improvements suggest that family members should continue to be engaged in interventions aimed at improving IYCF.

Just as social norms can influence an individual's attitudes (Cialdini et al., 1990), the effect of social norms on behaviours can be influenced as well. In a thorough review of social norms, Chung and Rimal (2016) highlight a variety of factors that can moderate the influence of norms on behaviours, including individual, behavioural and contextual attributes. Moreover, social norms alone cannot explain health behaviours (Cislaghi & Heise, 2019). Building on work demonstrating community‐based interventions that integrate addressing social norms alongside other social factors can be effective for behaviour change, Cislaghi and Heise (2019) propose the Dynamic Framework for Social Change, which includes four overlapping domains—institutional, material, social and individual. This is intended to be a practical framework that can be used to plan and implement health behaviour interventions that address a multitude of relevant behavioural drivers in addition to social norms. And while research in the health sciences field has begun to offer insights into the influence of social norms on health outcomes, most of the research studies to date have been conducted in high‐income countries, leaving much room to build an evidence base.

Furthermore, the importance of interpersonal communication on IYCF from health workers cannot be understated. Participants in our study often cited information from a health worker as a reason they agreed with advice related to EIBF and EBF, and health education related to child feeding was identified as a facilitator to achieving DD. Moreover, lack of knowledge was cited by some as a barrier to implementing optimal breastfeeding practices. Research has repeatedly shown that education of mothers of young children and other family members from health workers and through health facilities can facilitate optimal IYCF practices and that the lack thereof can do just the opposite (Balogun et al., 2015; Bazzano et al., 2017). Notably, results from our impact evaluation of the A&T intervention in Nigeria show increased odds of achieving EIBF (Kaduna and Lagos), EBF (Kaduna only) and minimum DD (Kaduna only) if mothers were exposed to interpersonal communication at a health facility or the intervention's mass media (Flax et al., 2022). Moreover, a study in Cambodia demonstrated that a lack of attendance at prenatal breastfeeding classes by fathers was a barrier to EBF (Sasaki et al., 2010). It is also clear, however, that adequate knowledge cannot power behaviour change by itself. For example, participants in this study cited having enough money to buy sufficient amounts of nutritious foods for pregnant and lactating women as well as children as an important factor for achieving optimal IYCF behaviours. The socioecological model acknowledges the role that societal conditions (e.g., economic, educational and social policies that can impact livelihoods and decision‐making) play in influencing behaviour change, and thus interventions should continue focusing on this crucial aspect as well.

While we have argued that understanding the perspectives of fathers and grandmothers is important, we acknowledge that their perspectives should not supersede those of mothers. In Ghana, Dumbaugh et al. (2014) found fathers were interested in being more involved with newborn care, whereas mothers were hesitant to relinquish this responsibility. Engaging key influencers in a way that caters to their own perceptions of their roles, if different from or in opposition to the mother's understanding of or preference for their role, has the potential to create unintended negative consequences on child feeding and women empowerment, both of which have been areas of concern in the literature and among programme implementers. Research from Northern Malawi and Zambia, however, demonstrates how designing an intervention to be sensitive to these issues can improve intrahousehold relationships, decrease familial conflict and improve child growth (Bezner Kerr et al., 2010; Kumar et al., 2018; Satzinger et al., 2009). Moreover, areas having harmonisation in beliefs should be leveraged, such as the common position grandmothers and elders hold as trusted sources of information because of their experience.

This study has several strengths. First, we collected data from multiple types of IYCF influencers across multiple time points of an intervention, thus capturing a thorough view of relevant sentiments. The quantitative data from the impact evaluation within which this study was nested provide additional context to our results. Many of the data collectors participated in each round of data collection, contributing to continuity and familiarity with the study and communities. The A&T intervention and overall impact evaluation were grounded in the socioecological model and had a theory of change that was tested in other countries. Furthermore, several Nigerians were involved in data analysis and reviewed the results, which enhanced interpretations of the data. The main limitations of this study were how analysis methods varied slightly from baseline to midline and endline and the inclusion of different analysis team members across time points. We mitigated these potential limitations by having the same person (CHS) manage data analysis teams across all time points, using the same codebooks across time points (only evolving as the data required) to maintain consistency, and meeting with team members regularly during the coding and analysis process to again maintain consistency and ensure a shared understanding of coding and analysis guidelines.

## 5 CONCLUSION

In conclusion, researchers in various contexts should continue to explore IYCF attitudes, beliefs and social norms from the perspectives of mothers of young children and key influencers because it will contribute more diverse perspectives and allow for the design of more holistic and effective IYCF interventions and programmes and support for families. We encourage researchers to publish their findings related to IYCF influencers in peer‐reviewed publications so that the results can be more accessible and widely used for planning IYCF interventions, programmes and communication strategies, which will further enhance the impact of such studies. There are several areas future research can focus on. Future research should include mothers, fathers and grandmothers in the same households to better understand family dynamics and how IYCF interventions targeted at different family members affect them (Allotey et al., 2022). Future research should also focus on understanding attitudes, beliefs, social norms *and* roles of child nutrition influencers to enhance intervention effectiveness. Finally, future research in LMICs should continue to include social norms as a construct for exploration to continue to build the evidence base for social norms and health behaviour in these contexts.

## AUTHOR CONTRIBUTIONS

Valerie L. Flax designed and oversaw the study, developed the data collection instruments and provided significant input to the manuscript during draughting. Courtney H. Schnefke contributed to the data collection instruments, managed data analysis across all time points and wrote the manuscript. Fred Ubanmhen oversaw data collection across all time points. Obinna Daniel contributed to data analysis at baseline. Kathryn E. L. Grimes contributed to data analysis at midline. Diana Allotey, Emily R. Seiger and Olujide Arije contributed to data analysis at endline. All authors contributed to the interpretation of the data, edited the manuscript and approved its final contents.

## CONFLICT OF INTEREST STATEMENT

SA and SB are or were employed by Alive & Thrive at the time of the study. SA was involved in the design, implementation and monitoring of the intervention but not in data collection or analysis for this study. SB was involved in the implementation and monitoring of the intervention but not in data collection or analysis for this study. All other authors have no conflicts of interest to declare.

## Data Availability

The data that support the findings of this study are available on request from the corresponding author.
